# The effect of cytosorb® application on kidney recovery in critically ill patients with severe rhabdomyolysis: a propensity score matching analysis

**DOI:** 10.1080/0886022X.2023.2259231

**Published:** 2023-09-20

**Authors:** Caroline Gräfe, Uwe Liebchen, Antonia Greimel, Nils Maciuga, Mathias Bruegel, Michael Irlbeck, Lorenz Weidhase, Michael Zoller, Michael Paal, Christina Scharf

**Affiliations:** a Department of Anesthesiology, LMU hospital, Munich, Germany; b Institute of Laboratory Medicine, LMU hospital, Munich, Germany; c Medical Intensive Care Unit, University Hospital Leipzig, Leipzig, Germany

**Keywords:** Rhabdomyolysis, myoglobin, kidney replacement therapy, blood purification, kidney recovery, cytosorb®

## Abstract

Severe rhabdomyolysis frequently results in acute kidney injury (AKI) due to myoglobin accumulation with the need of kidney replacement therapy (KRT). The present study investigated whether the application of Cytosorb® (CS) led to an increased rate of kidney recovery in patients with KRT due to severe rhabdomyolysis. Adult patients with a myoglobin-concentration >10,000 ng/ml and KRT were included from 2014 to 2021. Exclusion criteria were chronic kidney disease and CS-treatment before study inclusion. Groups 1 and 2 were defined as KRT with and without CS, respectively. The primary outcome parameter was independence from KRT after 30 days. Propensity score (PS) matching was performed (predictors: myoglobin, SAPS-II, and age), and the chi^2^-test was used. 35 pairings could be matched (mean age: 57 vs. 56 years; mean myoglobin: 27,218 vs. 26,872 ng/ml; mean SAPS-II: 77 vs. 76). The probability of kidney recovery was significantly (*p* = .04) higher in group 1 (31.4 vs. 11.4%, mean difference: 20.0%, odds ratio (OR): 3.6). Considering patients who survived 30 days, kidney recovery was also significantly (*p* = .03) higher in patients treated with CS (61.1 vs. 23.5%, mean difference: 37.6%, OR: 5.1). In conclusion, the use of CS might positively affect renal recovery in patients with severe rhabdomyolysis. A prospective randomized controlled trial is needed to confirm this hypothesis.

## Introduction

Rhabdomyolysis occurs frequently in intensive care unit (ICU) patients, resulting in elevated myoglobin and creatinekinase (CK) concentrations in the blood [1]. There are different causes for the disintegration of the skeletal muscle, e.g., polytrauma, sepsis, hemorrhagic shock, or medication toxicity [2,3]. The accumulation of myoglobin can lead to an acute kidney injury (AKI) up to the requirement of dialysis. High volume turnover as well as forced alkaline diuresis might be two opportunities to prevent patients of the necessity of kidney replacement therapy (KRT) [4]. However, both methods are only objective when diuresis is preserved.

The degradation of CK occurs by organ-independent degradation in patients’ blood. In contrast, myoglobin is excreted renally [5]. The elevation of myoglobin can lead to kidney function impairment and kidney parenchymal damage through various pathophysiological mechanisms and its concentration correlates with the development of AKI [6]. Myoglobin accumulation in patients with kidney impairment can result in potentially permanent kidney failure due to the damage of the tubular epithelial cells in the kidney [7]. The problem is that myoglobin clearance decreases rapidly in patients with anuric kidney failure, but its extracorporeal elimination with standard dialysis membranes is not effective due to the high molecular weight of myoglobin of approximately 17 kDa [8].

Various methods for sufficient extracorporeal myoglobin elimination have been introduced into clinical routine in recent years. On the one hand, high cutoff (HCO) dialyzers and high permeability dialysis membranes offer the opportunity to remove medium-sized molecules such as myoglobin [9,10]. Its intermittent use seems to be preferable in this context [11]. Similarly, plasmapheresis and continuous veno-venous hemofiltration (CVVH) can be used to achieve myoglobin elimination [12,13].

Another method for forced extracorporeal myoglobin elimination is the use of the cytokine adsorber Cytosorb^®^ (CS) (CytoSorbents Europe, Berlin, Germany). It was initially approved for the adsorption of cytokines but has also been licensed for the removal of myoglobin since 2019 [14]. Especially hydrophobic substances with a molecular weight of <60 kDa are bound to small beads, and a large adsorption capacity seems possible due to the presence of a surface area of 45,000 m^2^ [14]. In addition to case reports [15,16], an analysis from our group demonstrated a reduction in myoglobin in patients’ blood after the application of CS [17].

It is unclear for any of the above-mentioned procedures whether their use only affect laboratory parameters or if they improve patients’ outcome. In particular, kidney recovery from dialysis through the use of CS has not yet been investigated. To address this question, the effect of CS application on the primary outcome variable ‘kidney recovery at day 30’ – defined as independence from intermittent or continuous KRT following dialysis-requiring AKI – was investigated in patients with severe rhabdomyolysis using a propensity score (PS) matching analysis.

## Materials and methods

### Study setting

This was a monocentric, PS matching study, investigating the effect of CS therapy in critically ill patients with severe rhabdomyolysis. Patients treated between 2014 and 2021 at the anesthesiologic ICUs at LMU hospital in Munich were included. CS therapy was initiated or avoided according to the responsible physicians. Data from two clinical trials were evaluated to investigate the primary outcome variable ‘kidney recovery at day 30’. The local institutional review board approved the two studies (registration numbers 20-477 and 21-236, NCT04913298). Study one (20-477) was a retrospective analysis with the intention to evaluate myoglobin concentration in patients with rhabdomyolysis and renal failure. Study two (21-236) was a prospective trial to evaluate the adsorption capacity of CS in patients with severe rhabdomyolysis.

### Laboratory measurements and data collection

All laboratory parameters were determined at the Institute of Laboratory Medicine, LMU Munich, using routine instruments and assays. For data evaluation, demographic data, clinical, and laboratory variables were collected from the laboratory and patient information system.

### Study population

All patients with a myoglobin serum concentration > 10,000 ng/ml were screened retrospectively for study inclusion. Inclusion criteria were age > 18 years and the use of continuous kidney replacement therapy (KRT) in association to severe rhabdomyolysis. KRT was initiated by the attending physicians due to the AKI classification of the KDIGO [18]. All patients had an acute kidney injury stage 2 or 3, which was defined by both the creatinine clearance and the urinary output of the patient. High-flux dialysis (Fresenius Ultraflux® AV 1000S, surface area 1.4 m^2^, CVVHD CiCa^®^ or CVVHDF MultiBic® as indicated by the physicians) was used. Exclusion criteria were the necessity of KRT before ICU admission, chronic kidney disease > G3a (KDIGO–classification) before ICU admission [19], and CS treatment or ongoing KRT >24 h before study inclusion. Patients were divided into two groups: group 1 included patients with KRT (high-flux dialysis) and CS treatment and group 2 included patients with KRT (high-flux dialysis) but without CS therapy. The day of study inclusion (d0) was in both groups <24 h after initiation of CKRT and therefore equally for all patients. Patients allocated to group 1 got the first CS treatment at d0 (within 24 h after starting CKRT). Based on the clinical standard, patients were initially (∼2014–2017) treated infrequently with CS to reduce myoglobin, as it was not an established therapeutic modality at that time. In the course, the therapy was established in routine and most patients with severe rhabdomyolysis were treated with CS. The indication to start CS treatment was a myoglobin serum concentration >10,000 ng/ml. CS was installed post-dialyzer in the CKRT-circuit for 12–24 h per treatment session. The median duration of treatment were two days (IQR 2–3 d) and a median of 3 (IQR 2–4) CS were used per patient. The primary outcome measure was ‘kidney recovery at day 30’, which was defined as the independence of intermittent or continuous KRT on day 30, lasting for at least 7 d [20].

### Statistical analysis

Statistical analysis were performed using IBM SPSS statistics (Version 26.0., IBM Corp., Armonk, NY, USA). Differences in baseline parameters in both groups were detected using the chi*^2^*–test (nominal variables) or the *Welch*–test (other variables). PS matching (1:1) was performed to compare both groups. The Simplified Acute Physiology Score (SAPS) II, age, and myoglobin serum concentrations were used as predictors in the PS matching, as these parameters previously showed an effect on the recovery of kidney function or patients’ outcome [21–23]. The adjustment tolerance was <0.05, and the nearest neighbor method was used. The standardized difference ‘*d*’ (*d* = (mean A – mean B)/pooled standard deviation of both groups) was <10% after matching as a quality criterion [24]. The effect of CS treatment on kidney recovery on day 30 was investigated using the chi^2^–test. Differences in the myoglobin kinetics were detected using the Wilcoxon-test with associated samples.

## Results

### Selection of the study population

**Figure 1.**
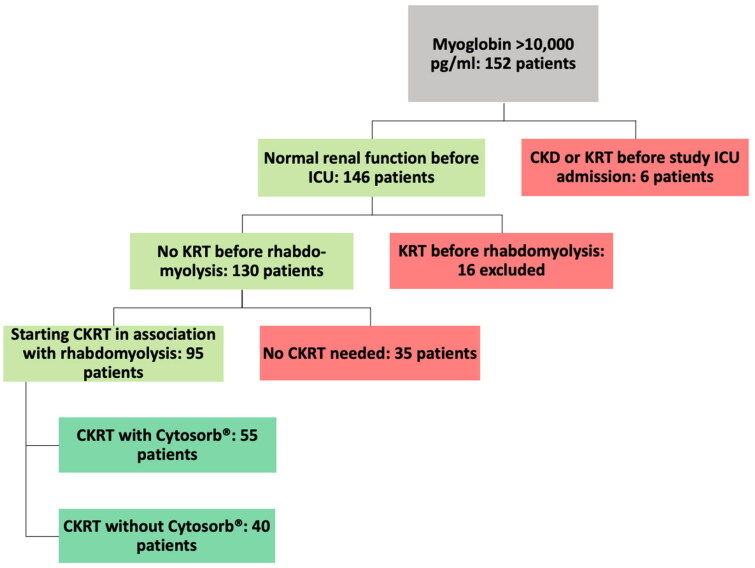
Selection of the study population. ICU: intensive care unit; CKD: chronic kidney disease; ®KRT: (continuous) kidney replacement therapy

Figure 1 illustrates the selection of the study population based on the above-mentioned inclusion and exclusion criteria.

### Demographic and clinical data

Table 1 shows the patient characteristics and laboratory measurements of the primary study population before PS matching.

**Table 1.** Patient characteristics before PS matching.

	Group 1: *n* (%) or mean [SD] or median {IQR}	Group 2: *n* (%) or mean [SD] or median {IQR}	*p*-value (*Welch /chi^2^*-test)
Number of patients	55 (100)	40 (100)	–
Age (years)	54 [16]	59 [18]	.21
Male	42 (76.4)	27 (67.5)	.34
BMI (kg/m^2^)	27.1 [6.8]	28.1 [6.1]	.48
ECMO therapy d0	16 (30.1)	11 (27.5)	.72
SAPS II d0	78 [16]	74 [18]	.21
SAPS II d0	78 {70, 88}	76 {63, 86}	
Mortality day 30	28 (50.9)	22 (55.0)	.70
Kidney recovery day 30	13 (26.6)	4 (10.0)	.07
Myoglobin d0 (ng/ml)	38,042 [43,776]	25,657 [19,876]	.08
Myoglobin d0 (ng/ml)	22,578 {12,391, 30,089}	18,814 {13,030, 28,396}	
CK d0 (U/l)	14,720 [28,270]	7194 [11,411]	.07
CK d0 (U/l)	3806 {2024, 12,607}	3818 {1687, 7644}	

BMI: body mass index; ECMO: extracorporeal membraneoxygenation; ICU: intensive care unit; SAPS: simplified acute physiology score; CK: creatine kinase; d0: study enrollment day.

### Propensity score matching

PS matching was performed as described in the methods section. 35 pairs were successfully matched due to the abovementioned criteria. Table 2 shows the patient characteristics after PS matching, the dialysis modality in both groups, and the reasons for the admission to the ICU. The standardized difference for the matched parameters age, SAPS II d0, and myoglobin d0 was 6.2, 5.6, and 4.3%, respectively. All patients were critically ill (mean SAPS II 76), frequently treated with ECMO-therapy (approximately 27%), and had a high 30-day mortality.

**Table 2.** Patient characteristics, dialysis modality, and reasons for the admission to the ICU after PS matching.

	Group 1: *n* (%) or mean [SD]	Group 2: *n* (%) or mean [SD]	*p*-value (Welch/chi^2^-test)
Patient characteristics			
Number of patients	35 (100)	35 (100)	–
Age (years)	57 [15]	56 [17]	.70
Male	26 (74.3)	23 (65.7)	.43
BMI (kg/m^2^)	27.6 [7.0]	28.0 [6.3]	.81
ECMO therapy d0	9 (25.7)	10 (28.6)	.79
SAPS II d0	77 [17]	76 [17]	.81
Mortality day 30	17 (48.6)	18 (51.4)	.81
Kidney recovery day 30	11 (31.4)	4 (11.4)	.04
Dialysis modalities			
Citrat/Heparin	12 (34.3)/23 (65.7)	19 (54.3)/16 (45.7)	.23
Blood flow (ml/min)	150 [68]	130 [56]	.46
Dialysate + (substituate) flow (ml/kg/h)	36 [18]	32 [13]	.29
CVVHD/CVVHDF	12 (34.3)/23 (65.7)	19 (54.3)/16 (45.7)	.23
Reasons for admission to the ICU			
Acute respiratory distress syndrome	9 (25.7)	10 (28.6)	
Polytrauma	5 (14.3)	4 (11.4)	
Septic shock	10 (28.6)	7 (20.0)	
Solid organ trans-plantation (lung or liver)	4 (11.4)	9 (25.7)	
Acute respiratory failure	3 (8.6)	0 (0.0)	
Aortic surgery	2 (5.7)	5 (14.3)	
Intoxication	2 (5.7)	0 (0.0)	

BMI: body mass index; ECMO: extracorporeal membrane oxygenation; CK: creatin-kinase; SAPS: simplified acute physiology score; d0: study enrollment day; CVVHD(F): continuous venovenous hemodialysis/hemodiafiltration.

Following Table 3 illustrates the main cause of rhabdomyolysis and the cause of death in both groups

**Table 3.** Main cause of rhabdomyolysis and death in both groups.

	Group 1: *n* (%)	Group 2: *n* (%)	*p*-value (chi^2^-test)
Main cause of rhabdomyolysis			
Polytrauma	5 (14.3)	4 (11.4)	.72
- Mortality	0 (0.0)	1 (25.0)	
- Independence of dialysis	3 (60.0)	2 (50.0)	
Viral infection (COVID-19 or influenza)	6 (17.1)	7 (20.0)	.76
- Mortality	4 (66.7)	4 (57.1)	
- Independence of dialysis	0 (0.0)	0 (0.0)	
Intestinal ischemia	4 (11.4)	3 (8.6)	.69
- Mortality	3 (75.0)	2 (66.6)	
- Independence of dialysis	1 (25.0)	0 (0.0)	
Intoxication/toxic side effect	3 (8.6)	2 (5.7)	.65
- Mortality	0 (0.0)	1 (50.0)	
- Independence of dialysis	1 (33.3)	0 (0.0)	
Post-resuscitation	2 (5.7)	3 (8.6)	.65
- Mortality	2 (100)	2 (66.6)	
- Independence of dialysis	0 (0.0)	0 (0.0)	
Arterial hypoperfusion e.g., compartment	4 (11.4)	6 (17.1)	.50
Mortality	2 (50.0)	3 (50.0)	
Independence of dialysis	1 (25.0)	1 (16.7)	
hypoxic respiratory failure	5 (14.3)	6 (17.1)	.75
- Mortality	3 (60.0)	4 (66.6)	
- Independence of dialysis	2 (40.0)	0 (0.0)	
Unclear	6 (17.1)	4 (11.4)	.50
- Mortality	2 (33.3)	1 (25.0)	
- Independence of dialysis	3 (50.0)	1 (25.0)	
Cause of death			
Liver failure	5 (29.4)	4 (22.2)	.72
Multi organ failure	5 (29.4)	5 (27.8)	1.00
Cardiac failure	2 (11.8)	4 (22.2)	.40
Septic shock	2 (11.8)	2 (11.1)	1.00
Cerebral bleeding	3 (17.6)	3 (16.7)	1.00

### Comparison of the matched study population

Table 4 shows different laboratory parameters in both groups.

**Table 4.** Laboratory parameters in both groups.

	Group 1: mean [SD]	Group 2: mean [SD]	*p*-value (Welch/chi^2^-test)
Myoglobin d0 (ng/ml)	27,218 [22,573]	26,872 [20,582]	.95
Myoglobin d1 (ng/ml)	25,704 [28,237]	29,032 [26,685]	
Myoglobin d2 (ng/ml)	19,646 [19,035]	29,323 [26,685]	
Myoglobin d6 (ng/ml)	4530 [3856]	5382 [5568]	
CK d0 (U/l)	9683 [11,697]	7856 [11,868]	.53
CK d1 (U/l)	12,499 [16,566]	9610 [13,636]	
CK d2 (U/l)	10,189 [15,752]	5880 [10,137]	
Lactate d0 (mmol/l)	6.2 [5.3]	6.1 [6.9]	.97
Creatinine d0 (mg/dl)	2.3 [1.6]	2.3 [1.5]	.89
Urea d0 (mg/dl)	87 [45]	89 [44]	.84
Sodium d0 (mg/dl)	4.5 [0.7]	4.9 [0.8]	**.03**
Bicarbonate d0 (mmol/l)	21.3 [5.5]	22.3 [4.7]	.45
Potassium d0 (mmol/l)	4.6 [0.5]	5.0 [0.9]	**.02**
Phosphate (mg/dl)	4.9 [1.8]	4.7 [1.6]	.33
eGFR (CKD-EPI) d0 (ml/min)	44 [27]	43 [27]	.85
eGFR (CKD-EPI) d30 in patients without dialysis (ml/min)	68 [43]	52 [22]	.29
Residual diuresis d0 (ml/24 h)	400 [660]	440 [730]	.80

**Figure 2.**
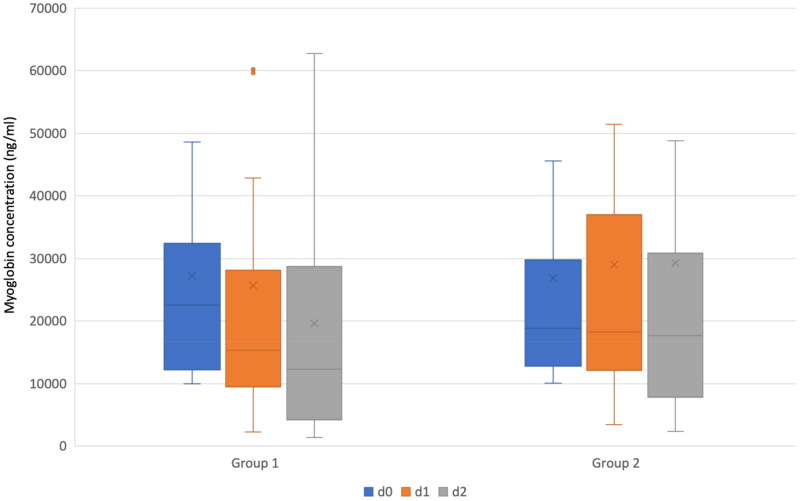
Myoglobin serum concentration (ng/ml) on days 0, 1, and 2 in patients treated with cytosorb^®^ (group 1) and those without cytosorb^®^ application (group 2). Group 1 included patients treated with Cytosorb^®^ and group 2 without Cytosorb^®^ application. The boxes of the boxplots represent the interquartile-range (IQR) and the line the median. Whiskers were limited to 1.5 times the IQR. The cross represents the mean. Blue boxplots represent day 0 (d0), orange ones day 1 (d1), and grey ones day 2 (d2).

The median myoglobin at d0 and d1 in patients allocated to group 1 was 22,578 (IQR: 12,391–30,089) and 15,373 (IQR: 9808–27,336) ng/ml with a significant reduction (*p* < .01). A significant decrease was also observed between d0 and d2 (median myoglobin d2 12,357 ng/ml (IQR: 4385–26,884); *p* = .03). The median myoglobin at d0 and d1 in patients allocated to group 2 was 18,814 (IQR: 13,030–28,396) and 18,261 (IQR 12,293–36,633) ng/ml with no significant reduction. There was also no significant decrease between d0 and d2 (median myoglobin d2 17,586 ng/ml, (IQR: 7985–30,265)). The median (IQR) CK in patients allocated to group 1 was on days 0, 1 and 2 3806 (2024–12,607), 5719 (2779–15,033), and 4400 (1605–9718) U/l with no significant change during the time. Similar results were observed in patients allocated to group 2 with a median (IQR) CK on days 0, 1, and 2 of 3818 (1687–7644), 4894 (1741–9058), and 2452 (1332–4232) with no significant change between days 0 and 1, but a significant reduction between day 1 and 2 (*p* < .01). Following Figure 2 illustrates the myoglobin concentration on day 0, 1, and 2 in both groups as box plots.

Kidney recovery on day 30 was observed in 31.4 and 11.4% of patients allocated to groups 1 and 2, respectively. It was significantly more frequent in patients allocated to group 1 compared to group 2 (*p* = .04, odds ratio (OR): 3.6, 95% confidence interval: 1.1–11.6). The mean risk reduction was 20.0%, and the number needed to treat was five. Termination of KRT was not observed in any of the patients who died prior to day 30. Following Table 5 shows different parameters that might affect kidney recovery in patients who survived until day 30 (group 1: 18 patients, group 2: 17 patients).

**Table 5.** Parameters of kidney recovery in survivors until day 30.

	Group 1: mean [SD]	Group 2: mean [SD]	*p*-value (Welch-test)
Duration until kidney recovery (days)	13.2 [7.3]	13.8 [7.9]	0.46
eGFR (CKD-EPI) d0 (ml/min)	47 [28]	48 [29]	0.48
eGFR (CKD-EPI) d7 (ml/min)	66 [45]	61 [40]	0.38
eGFR (CKD-EPI) d14 (ml/min)	69 [38]	63 [43]	0.66
eGFR (CKD-EPI) d30 (ml/min)	83 [41]	74 [47]	0.28
Duration of inotropes (days)	20 [9]	19 [11]	0.32
Duration of mechanical ventilation (days)	17 [12]	22 [11]	0.09

**Figure 3.**
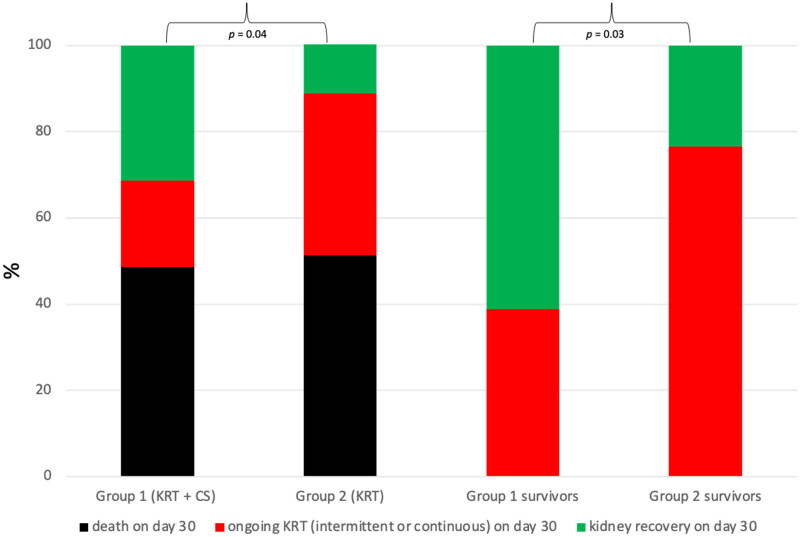
The proportion of patients (%) with kidney recovery, ongoing KRT or death on day 30. KRT: kidney replacement therapy; CS: Cytosorb^®^, kidney recovery was defined as independence of KRT on day 30, which then lasts for at least seven days. Kidney recovery was significantly more often observed in patients treated with dialysis + CS compared to dialysis alone.

Based on those patients who survived 30 days after study inclusion, kidney recovery with and without CS was 61.1 and 23.5%, respectively. It was also significantly more frequent in patients allocated to group 1 compared to group 2 (*p* = .03, OR: 5.1, 95% confidence interval: 1.1–9.6). The mean risk reduction was 37.5%, and the number needed to treat was 2.7. The following Figure 3 illustrates the percentage of patients with kidney recovery, ongoing KRT, and death on day 30 in both groups.

## Discussion

Severe rhabdomyolysis leads to the release of myoglobin and CK into the blood [25]. The need of KRT due to severe rhabdomyolysis is associated with an increased mortality in critically ill patients and its therapy remains challenging for responsible health-care professionals [26,27]. *Candela* et al. were able to show that the renal outcome in these patients correlates strongly with the myoglobin serum concentration [28]. If KRT is needed due to an anuric kidney failure, extracorporeal myoglobin elimination might be an approach to attempt the recovery of kidney function and to abbreviate the duration of continuous and intermittent KRT [29,30]. Therefore, dialyzers with larger cutoff values have been used successfully for several years [31,32], but data regarding efficacy in terms of patient outcome are completely lacking.

The presented study examined the effects of CS in critically ill patients with severe rhabdomyolysis and the necessity of continuous KRT on kidney recovery defined as independence from intermittent or continuous KRT on day 30. There was no significant difference in the baseline parameters of all patients (Table 1) before PS matching. Nevertheless, PS matching of the two groups was performed to minimize the influence of confounders and to achieve a higher degree of concordance of the data. Three predictors were used for matching: SAPS II as a predictor for the severity of the disease, patients’ age as a predictor to estimate the recovery from the underlying disease, and myoglobin as a predictor for the severity of rhabdomyolysis and renal toxicity. CK was not included, as it neither affects kidney function nor is eliminated renally or by CS. The studied population was extremely ill with a high SAPS II and 30-day mortality. However, severe rhabdomyolysis with the indication for CKRT affects severely ill patients and high mortality rates of up to 59% are described [33,34]. Furthermore, the different causes of rhabdomyolysis are typical for ICU patients. In particular, viral infections with influenza or COVID-19 are associated with severe rhabdomyolysis [34].

Thirty-five pairs were successfully matched with no relevant baseline differences (Table 2), demonstrating that the PS matching worked well. PS matching can be regarded as the gold standard to compare two groups in a retrospective dataset and it is regularly used for this purpose [24,35]. In the matched population, the probability of kidney recovery, defined as the end of KRT on day 30, was found to be significantly more likely with CS than without. The mean relative risk reduction was 20% and the number needed to treat was five. These new results do not exist for CS or for any other procedure that eliminates myoglobin so far. Furthermore, a significant decrease of myoglobin in the blood on days 1 and 2 was observed in patients treated with CS, whereas no significant change was detected in those with standard care (CVVHD or CVVHDF).

Considering only those patients who survived 30 days after study enrollment (group 1: 18 patients; group 2: 17 patients), a significantly more frequent recovery of kidney function was also observed in patients treated with CS. The mean risk reduction of ongoing dialysis-requiring kidney failure was 38%, corresponding to the number needed to treat of 2.7. However, no significant difference in 30-day mortality was observed between both groups (48 vs. 51%). This can be explained as all patients had other serious medical conditions and not only severe rhabdomyolysis (e.g., acute respiratory distress syndrome or solid organ transplantation). Treatment and prognosis of these diseases are likely unaffected using CS. However, if patients finally survive the severe disease, it is of high relevance whether chronic kidney failure requiring dialysis develops or not. Our results indicate that this risk might be reduced using CS. When using unspecific adsorption procedures such as CS, it must be noted that various substances can be adsorbed. In example: while there is no evidence of a relevant meropenem adsorption by CS [36], it was recently shown that more than 500 mg vancomycin are adsorbed in one CS treatment session [37]. Thus, it remains unclear whether it was the adsorption of myoglobin alone that contributed to a faster recovery of renal function or whether the adsorption of other metabolites by CS may also have contributed.

Kidney recovery is enormously important as well at the ICU as after surviving the critical disease. On the one hand, the venous catheter for dialysis is a source of infection that can lead to sepsis [38]. In addition, ongoing kidney failure requiring KRT is associated with an increased risk of cardiovascular complications and death [39]. Furthermore, the patients’ quality of life, which is significantly reduced by ongoing KRT, must be considered [40]. Last, ongoing dialysis treatment causes high costs and can lead to kidney transplantation, which is additionally associated with lifelong immunosuppression, resulting in an increased rate of infections. As myoglobin has direct toxic effects, extracorporeal removal seems reasonable [41]. Indeed, not only CS but also HCO dialyzers or the use of CVVH with high blood flow are suitable for this purpose [31]. Whether and which method has the best outcome is not clear yet and no clear treatment strategy can be given.

Due to the retrospective and monocentric study approach, the present work has several limitations. Although PS matching allows for the comparison of two populations, it is not comparable to a randomized controlled trial. This should follow in the future to further investigate these new findings in a larger population. Furthermore, the population of 70 patients is relatively small, which limits the power of the study. However, it must be noted that no data concerning this research question are available to date. In addition, the observation period was limited to 30 days; it is unclear whether there was recovery from dialysis in some patients later. Moreover, the cause of the necessity of CKRT is often multifactorial in critically ill patients. This could have implications for the results of the study, though the underlying diseases of the patients were similarly distributed in both groups. Finally, CS was used in routine clinical practice and, thus, not under controlled study conditions. The number and duration of CS treatments was at the discretion of the attending physicians. Again, a prospective study would be helpful in this purpose.

## Conclusions

In conclusion, the use of CS might positively affect renal recovery in patients with severe rhabdomyolysis and dialysis-requiring acute kidney injury. The gained findings are suitable to establish the hypothesis that the application of CS might be beneficial in those patients. A prospective randomized controlled trial is needed to confirm this hypothesis.

## Data Availability

All data have been included to the manuscript.
